# Chitosan-Based Materials for Peripheral Nerve Repair—New Pre-Clinical Data on Degradation Behavior at the Nerve Repair Site and Critical Opinion on Their Translational Impact

**DOI:** 10.3390/ijms26031214

**Published:** 2025-01-30

**Authors:** Giulia Ronchi, Christina Ackva, Federica Fregnan, Federica Zen, Luisa Muratori, Alessandro Crosio, Jennifer Metzen, Kirsten Haastert-Talini

**Affiliations:** 1 Department of Clinical and Biological Sciences & Neuroscience Institute Cavalieri Ottolenghi (NICO), University of Torino, 10043 Orbassano, Italy; federica.fregnan@unito.it (F.F.); federica.zen@unito.it (F.Z.); luisa.muratori@unito.it (L.M.); 2 KeriMedical, Route des Acacias, 45a, 1227 Genf, Switzerland; 3 Medovent GmbH, Friedrich-Koenig-Str. 3, 55129 Mainz, Germany; 4 UOC Traumatology-Reconstructive Microsurgery, Department of Orthopedics and Traumatology, CTO Hospital, 10126 Turin, Italy; alessandro.crosio@unito.it; 5 Institute of Neuroanatomy and Cell Biology, Hannover Medical School, Carl-Neuberg-Str. 1, 30625 Hannover, Germany; metzen.jennifer@mh-hannover.de (J.M.); haastert-talini.kirsten@mh-hannover.de (K.H.-T.); 6 Centre for Systems Neuroscience (ZSN), 30559 Hannover, Germany

**Keywords:** chitosan nerve guide, degradation in vivo, nerve regeneration

## Abstract

Before clinical approval of chitosan nerve conduits (CNCs) less than 10 years ago, substantial material degradation was not reported from pre-clinical research. The current study investigated the biodegradation of two different CNC variants in the median nerve model. In adult rats, 10 mm long CNCs were secured covering end-to-end repair sites. After 2, 6, 12, and 18 months, the implantation sites were inspected, and samples harvested. Histology was performed in order to analyze local immune response or foreign body tissue reaction around the devices or within nerve samples. Further, the number of myelinated nerve fibers and the condition of the chitosan material was evaluated. Data confirmed that different CNC variants did not induce tissue reaction or negatively impact the condition of the regenerated nerve. At late time points, some specific variants were demonstrated to have resulted in encapsulated material debris in the vicinity of the original implantation side. The reported degradation behavior resembles recent clinical reports and indicates that implantation sites for CNCs need to be chosen carefully. Nerve regeneration itself is undisturbed, but narrow implantation sites should be avoided for eliminating the risk of foreign body sensation with immunologically inert material degradation.

## 1. Introduction

Chitosan is a natural biopolymer that offers a variety of beneficial properties for regenerative medical approaches [1]. Advances in material science have brought up the possibility to produce transparent, collapse-stable, and sewable nerve guidance channels from chitosan for the repair of transected peripheral nerves. Chitosan nerve conduits (CNCs) have been researched intensively in pre-clinical models and have finally been approved for clinical use less than 10 years ago. The approval was based on relevant and comprehensive pre-clinical data mainly derived from the rat sciatic nerve injury and repair model [2]. Pre-clinical studies did not only focus on the outcome of nerve repair in comparison to CNCs with the gold standard autologous nerve graft [3], but specifically considered the aspect of their degradability [4]. A comprehensive amount of pre-clinical data was provided on the low immunogenicity of the implanted CNCs and only minimal scarring at the outer sides of the material in vivo [3]. In addition to nerve gap repair, CNCs have also been promoted as a protective cover for the tensionless end-to-end epineurial coaptation of transected nerve ends for enhancing nerve regeneration [2]. A clinical trial investigating this approach in repaired digital nerves used CNCs (Reaxon^®^ Direct 1.0, KeriMedical, Geneve, Switzerland) with different wall thicknesses [5,6].

Reaxon^®^ Direct 1.0 came to the market in 2016 as the shorter variant of the original Reaxon^®^ Nerve Guide. The product variants were developed to bridge peripheral nerve defects up to 26 mm (Reaxon^®^ Nerve Guide, Kerimedical, Genf, Switzerland) or 10 mm (Reaxon^®^ Direct 1.0, Kerimedical, Genf, Switzerland), respectively. The product was developed to protect and allow the regenerating nerve to fully regenerate and mature. Therefore, a minimum stability of the tube of 6 months was required. Reaxon^®^ Direct 1.0, in its initially approved form, has shown very slow degradation in the animal studies [3].

As the result of a clinical study showing the superiority of protecting a direct end-to-end suture by a Reaxon^®^ tube, in comparison to end-to-end suture alone, Reaxon^®^ Direct 1.0 was mostly used for this indication [6]. Most injuries treated with Reaxon^®^ Direct were located in fingers and wrists. For this indication, the long-term stability of the tube is not required and a faster degradation without the risk of secondary damage to the nerve or peripheral soft tissues would be highly appreciated by the users. Reaxon^®^ Direct 1.0 was a spin-off product from the Reaxon^®^ Nerve Guide. The preclinical studies performed to investigate the regenerative properties of the CNCs used observation periods of up to 5 months after implantation of the nerve guides into the acutely or chronically transected rat sciatic nerves, and no obvious degradation was detected during this period [3]. In addition, a long-term study analyzing regeneration and nerve guide properties over a period of up to 76 weeks did not reveal significant degradation of the CNCs [4]. Therefore, slow degradation in human patients was predictable. Within an unpublished short preclinical study, novel variants of the Reaxon^®^ Direct 1.0 device have been tested in order to detect a variant that would be stable for sufficient time to enable a protective effect on direct sutures and that would very likely, in human patients, have a degradation time of about 6–12 months. From the results of this previous study, a variant of DA9/WT50 (degree of acetylation 9/Wall thickness 50) was selected and introduced to the clinic as Reaxon^®^ Direct 2.0 (Kerimedical, Genf, Switzerland). The Reaxon^®^ Direct 2.0. is further investigated in the study presented here. We analyzed the degradation behavior in a preclinical setting and compared the Reaxon^®^ Direct 2.0 variant with a variant comprising a higher degree of acetylation (DA13) and an equal wall thickness. The higher degree of acetylation was selected because it should guarantee a more rapid biodegradation in comparison to DA9. With our study, we intended to exclude that the faster degradation process activates an increased immune response or foreign body tissue reaction. We considered that the latter, in particular, would increase the risk of secondary entrapment of the nerve due to possible secondary fibrosis around the wrap CNCs. Nerve entrapment could again impair nerve function. We considered the median nerve model more appropriate for the current study than the sciatic nerve model. When sutured to the median nerve, the CNCs are placed in a region that is more mobile than in the sciatic nerve [7], thus modeling better the situation when the device is sutured to digital nerves in human patients.

The Reaxon^®^ Direct 2.0 variant (DA9/WT50), whose intended use is limited to end-to-end suture protection, has been available on the market since January 2020, and has replaced the initially developed slowly degrading version. The current study was continued for up to 18 months after implantation surgery and we plan to be observing the degradation behavior until (almost) complete biodegradation. Observations from clinical use of Reaxon^®^ Direct 1.0. and 2.0 results have been published recently [8] and will be discussed below in view of our study that has been conducted in parallel and now adds to our current understanding of the biodegradation of the chitosan based Reaxon^®^ nerve guide products.

Here, 10 mm long conduits of different variants of Reaxon^®^ Direct 2.0 were pulled over the end-to-end suture site repairing the transected rat median nerve and fixed to both nerve stumps, each with a single epineurial suture. Control nerves were likewise transected and end-to-end repaired but left uncovered. At the time of 2 months, 6 months, 12 months, and 18 months after implantation, the animals were sacrificed for inspection of the implantation site, remaining chitosan material, and sample harvest. Median nerve samples were collected from both the segment enwrapped by the device and a segment distal to it. These samples have then been processed for histological analysis of the local immune response or foreign body tissue reaction, as well as for histomorphometric analysis of nerve tissue.

The specific aims of our study were as follows:

- Aim (1): to observe the degree of material degradation and its subsequent behavior. In order to observe this, we macroscopically evaluated the fate of the material and the properties of the degraded material residues (appearance, haptic) at the different explantation time points, thereby determining the degree of degradation of the conduits.
- Aim (2): to compare uncovered and CNC covered end-to-end repaired median nerve samples, with regard to stereo-morphometrical parameters in order to understand if material degradation interferes with axonal regeneration.
- Aim (3): to compare uncovered and CNC covered nerve tissue and surrounding epineurium for the presence of immune reaction or foreign body response related to material degradation.

## 2. Results

In this study we basically compared two variants of Reaxon^®^ Direct 2.0, the proper variant DA9/WT50 and the variant D13/WT50, both of which are used to cover the end-to-end nerve repair site. As a control, median nerves were transected and end-to-end sutured for repair but left uncovered. The experimental groups are summarized in Table 1.

A total of 38 animals were introduced into this study and all procedures were performed in accordance with the Italian Animal Welfare Legislation and the principles of the Basel Declaration and recommendations of Directive 2010/63/EU and were approved by the Ethic Experimental Committee of the University of Torino and registered under the Ministry of Health project number 864/2016.

During the post-implantation observation period, the animal health status as well as the surgical sites were regularly checked. The shine of the rat fur, normal reactivity, and general health were documented, and no signs of pain, discomfort, or apathy were reported. Likewise, the aspect of the surgical wound, and the respectively healed area, appeared normal and without signs of inflammation.

One animal belonging to Group 2 of the 12-month trial died before the end of the trial. This death did not appear to be related to the implantation of the conduit.

Four animals belonging to Group 1 and two animals belonging to Group 2 of the 18-month trial died before the end of the trial. Again, their deaths did not appear to be related to the implantation of the conduit, but rather to the age of the animals.

In the following subsections we will report data specific to the above mentioned aims of this study.

### 2.1. Macroscopic Assessment of the Implantation Site over Time Confirms Rapid Degradation of DA13/WT50 Variant

Degradation was quantified through visual inspection, using specific criteria to assess the material’s progression over time. Initially, we inspected the grafted material for any type of deformation or flattening, which indicated the first changes in the shape and structure of the CNCs. As degradation progressed, we recorded the formation of cracks on the surface of the CNCs, which signified further deterioration. The material was then examined for fragmentation, starting with breaking into larger pieces and progressing to the formation of smaller fragments. In the final stages of degradation, the material was evaluated for the formation of a large number of tiny pieces. In addition to these structural changes, we also assessed the haptic properties of the material, including its firmness, softness, and flexibility to provide further insight into the extent of degradation.

Two months after implantation, DA9/WT50 tubes (n = 4) were not degraded and had a clear, transparent appearance and a soft, flexible haptic. However, two of the CNC tubes were found to be broken on the proximal end, where they had likely been manipulated with some force during implantation.

DA13/WT50 CNCs were substantially degraded and broken into soft pieces that had maintained a clear, transparent appearance. Occasionally, degraded chitosan pieces were found encapsulated in pocket-like structures formed by connective tissue with only loose contact to the epineurium of the nerve.

All end-to-end-suture sites covered by the CNCs subjectively appeared with a slightly increased vascularization and slightly increased diameter, as compared to the uncovered nerve tissue outside the tubes. The end-to-end sutured uncovered repair sites also appeared slightly thickened and more vascularized than adjoining nerve segments.

Six months after implantation, DA9/WT50 conduits had retained a clear, transparent appearance and a soft, flexible haptic. Initial signs of biodegradation were recorded, with most CNCs exhibiting a longitudinal fracture and some showing a slight fragmentation.

DA13/WT50 tubes were almost fully degraded: in two cases, only very small fragments were still visible, while in one animal the conduit was completely degraded and no fragments were visible macroscopically; in the fourth animal, one half of the previous conduit was still present. Fragments that could be harvested were very soft and brittle, making it impossible to separate them from the surrounding connective tissue without causing further fragmentation.

The macroscopic appearance of the end-to-end-suture sites, either covered by the CNCs or left uncovered, was similar to the two-month time point, subjectively more vascularized and a bit increased in diameter.

Twelve months after implantation, DA9/WT50 conduits had retained their overall shape but were visibly flattened in situ. With dissection, they were either demonstrated to have been broken into two longitudinal larger pieces or to be even more brittle and broken up into smaller pieces upon manipulation. In cases where the CNCs had already broken in a longitudinal direction, thin layers of connective tissue had been formed, extending from the epineurium into the CNC-clefts (three of the five). Interestingly, in two of the five implantation sites, DA9/WT50-CNCs did not demonstrate obvious degradation but, when they broke off during manipulation, some chitosan pieces encapsulated in connective tissue pockets at the outside of the devices were also occasionally detected. The CNC-pieces were found to be soft and appeared clear to yellowish.

DA13/WT50 conduits showed disintegration into uncountable high numbers of small pieces enclosed into connective tissue formed around the surgical site. In two of the four specimens, a thickened connective tissue or a separated connective tissue capsule containing a high amount of chitosan pieces and located in some vicinity to the original surgery site were detected, respectively.

The uncovered control end-to-end-suture sites still presented with a slightly increased diameter around the suture, while vascularization was equal along the complete inspected length of the nerve.

Similar to the findings of the 12-month examination time point, 18 months after implantation, DA9/WT50 devices appeared to still have maintained their overall shape, although they were noticeably flattened in situ. With dissection, the DA9/WT50-CNCs were demonstrated to be broken into two longitudinal larger pieces that further broke up into more pieces upon manipulation. Encapsulated pieces of DA9/WT50-CNC material were not found in any of the respective implantation sites that have been available for inspection after 18 months. CNC pieces were found to be soft and had a clear to yellowish appearance.

DA13/WT50 devices showed disintegration into high numbers of small pieces enclosed into a thickened connective tissue around the surgery site.

Overall, it subjectively appeared to the examiner that the material was less in overall amount, e.g., thickness in DA9/WT50 or amount of pieces in DA13/WT50. This observation, however, could not be verified with quantitative measures.

The uncovered control end-to-end-suture sites presented as described above for the 12-month time point.

We can further report that over all time points, although not quantified with objective methods, the pieces of degraded CNC material could not be described as solid or edgy.

### 2.2. Histomorphometrical Analysis of Semi-Thin Nerve Cross Sections Distal to the CNC Revealed No Impairment of the Nerve Regeneration Process

In the 2-month trial and in the 18-month trial, one sample each, representing the uncovered control condition or the DA9/WT50 condition, respectively, was not properly fixed and could therefore not be analyzed. Because of the death of one animal from Group 2 in the 12-month trial and four animals from Group 2 and two animals from Group 1 in the 18-month trial, the number of the respective specimens available for analysis was reduced. Table 2 summarizes the number of specimens analyzed in each trial.

**Figure 1:**
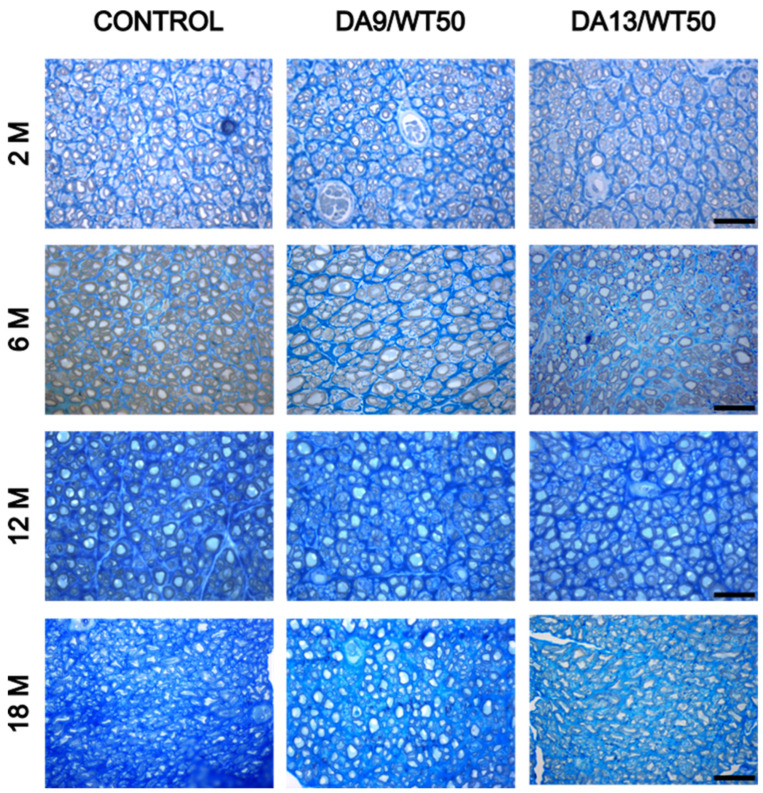
Representative high-magnification light photomicrographs of toluidine blue-stained semi-thin sections of regenerated median nerves distal to the CNCs at 2, 6, 12, and 18 months after injury and end-to-end repair for each condition. Successful regeneration of the median nerve is evident across all conditions and time points. Scale bars = 20 μm.

Histomorphometrical analysis of the regenerated median nerve distal to the CNCs was performed to understand whether material degradation interferes with axonal regeneration. Semi-thin toluidine-blue stained cross sections were analyzed by high resolution light microscopy. One representative picture for each sample is shown in Figure 1.

**Figure 2:**
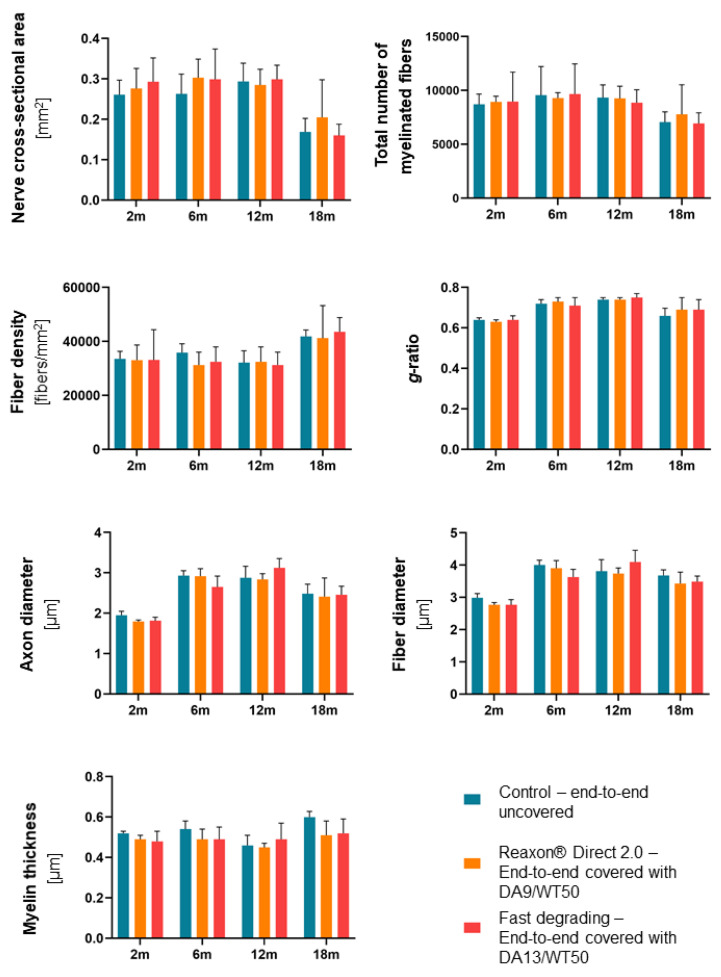
Results of the histomorphometrical analysis on the distal portion of the CNCs at 2, 6, 12, and 18 months after injury revealed no statistically significant differences between the experimental groups at any time point. Data are expressed as a mean ± SD. Statistical analysis: one-way ANOVA followed by Bonferroni’s post hoc test.

Figure 2 summarizes the data derived from the analysis of the following parameters: nerve cross-sectional area, total number of myelinated fibers, density of myelinated fibers/mm^2^, *g*-ratio, axon diameter, myelinated fiber diameter, and myelin thickness. No significant differences among the three groups were found for any of the analyzed parameters at any time point.

### 2.3. Histological Analysis of the Thickness of the Epineurium Revealed Highest Values in the Reaxon^®^ 2.0—DA9/WT50 Group

**Figure 3:**
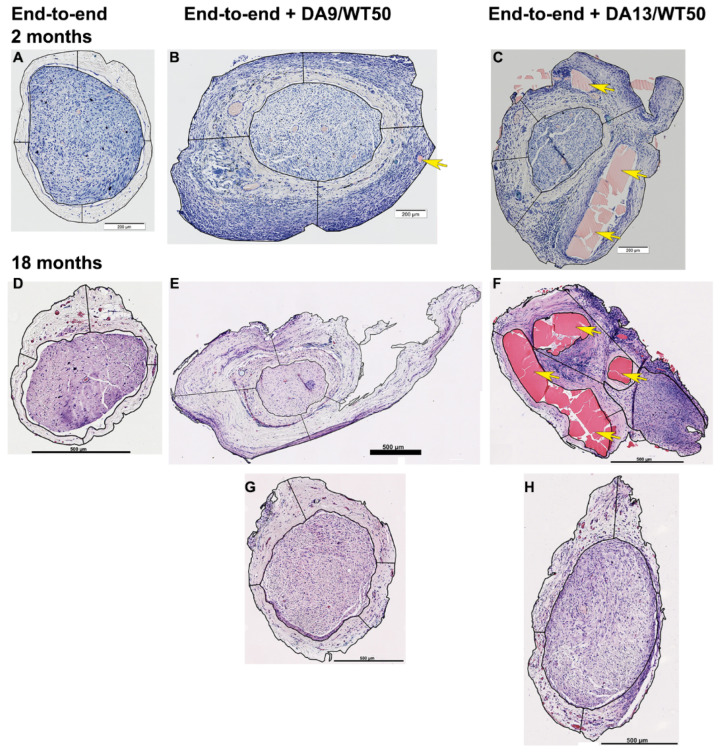
Representative overview of Giemsa-stained cross sections through the nerve and its epineurium harvested from inside the device at 2 months (**A**–**C**) and 18 months (**D**–**H**) after implantation. (**A**,**D**) End-to-end uncovered. (**B**,**E**,**G**) Reaxon^®^ 2.0—end-to-end covered with DA9/WT50. (**C**,**F**,**H**) Fast degrading—end-to-end covered with DA13/WT50. The inner black circular line represents the border line encircling the nerve area, the outer black circular line represents the border line encircling the epineurium. Straight lines represent the randomly chosen locations where distances have been measured between the encircling lines for calculating the mean epineurial thickness for each section. Yellow arrowheads point to pinkish amorphous material in (**B**,**C**,**F**), representing encapsulated degrading chitosan material within the epineurium. The latter is most prominent in samples from the fast degrading group (DA13/WT50 variant). Scale bars: in (**A**–**C**) = 200 µm; in (**D**–**H**) = 500 µm.

Cross-sections through the nerve and its epineurium (connective tissue underneath the CNC cover where appropriate) were stained according to the Giemsa protocol for clearly distinguishing the central nerve tissue and the outer epineurium. Representative photomicrographs used for this type of analysis at the 2-month and 18-month time points are shown in Figure 3. Representative photomicrographs from all time points are provided in the Supplementary Material (Figures S1–S4).

Two months after implantation, the epineurium formed under DA9/WT50-CNCs was significantly thicker (406 ± 90 µm, N = 4) than the one formed around an uncovered nerve (102 ± 24 µm, N = 4) after end-to-end suture. Increase of epineurial thickness was also detectable under DA13/WT50-CNCs (239 ± 47 µm, N = 4), but the increase was not statistically different from the control condition epineurium. Inclusions of degrading CNC-material were mainly found in the DA13/WT50 group at this time point (Figure S1).

Six months after implantation, no significant differences were detectable between the groups regarding epineurium thickness, either near the suture site or beneath the CNC. The following values (mean ± standard deviation) were determined: uncovered nerve (202 ± 126 µm, N = 4); DA9/WT50-CNC-group (396 ± 119 µm, N = 8); DA13/WT50-CNC-group (267 ± 122 µm, N = 4). Two of the analyzed samples from the uncovered group displayed a visibly increased amount of blood vessels within their epineurium (see Supplementary Figure S2). The epineurium underneath the DA9/WT50-CNCs showed inclusions of numerous bigger or few smaller CNC-material pieces in 50% of the evaluated specimen (see Supplementary Figure S2). Analyzed sections from the DA13/WT50 group showed epineurial inclusions of degrading CNC-material in three out of four specimens (Figure S2).

Twelve months after implantation, no significant differences were again detectable between the groups. Subjectively, however, CNC-covered groups tended to display a slightly thickened epineurium in comparison to the uncovered group (similar to what has been described for the 2-month time point). Unfortunately, standardized thickness quantification was not possible for all samples because the epineurium appeared to be spared on the sections and pieces might have been lost during histological preparation. The following values (mean ± standard deviation) were determined: uncovered nerve (211 and 163 µm, N = 2; more samples were not available for evaluation); DA9/WT50-CNC-group (332 ± 72 µm, N = 9); DA13/WT50-CNC-group (347 ± 227 µm, N = 3; more samples were not available for evaluation). In two of the nine DA9/WT50-specimens, a single big piece of degrading CNC material was visible within their epineurium (Figure S3).

Eighteen months after implantation, no significant differences were detectable between the groups, although end-to-end sutured nerves covered with CNCs subjectively always display a slightly thickened epineurium upon dissection in comparison to the uncovered end-to-end sutured nerves (similar to what has been described for all previous examination time points). One sample of the uncovered and one of the DA9/WT50 group was not available for evaluation because the respective animal died before sample harvest. The following values (mean ± standard deviation) were determined: uncovered nerve (98 and 105 µm, N = 2); DA9/WT50-CNC-group (284 ± 78 µm, N = 6); DA13/WT50-CNC-group (229 ± 139 µm, N = 4). During dissection it appeared that degrading CNC-material was easier to separate in the DA9/WT50-group specimen, whereas it still could be detected to be encapsulated within the epineurium in the evaluated DA13/WT50 sections (Figure S4).

Over all analyzed time points, the thickness of the epineurium underneath the CNC-cover was regularly thickest in the DA9/WT50 group, but the parameter did not change significantly over time in any of the groups.

### 2.4. Immunohistological Analysis and Quantification of Activated Macrophages (ED1-Immunopositive Cells) Revealed Pronounced Foreign Body Reaction in the Epineurium Underneath DA9/WT50 Covers

**Figure 4:**
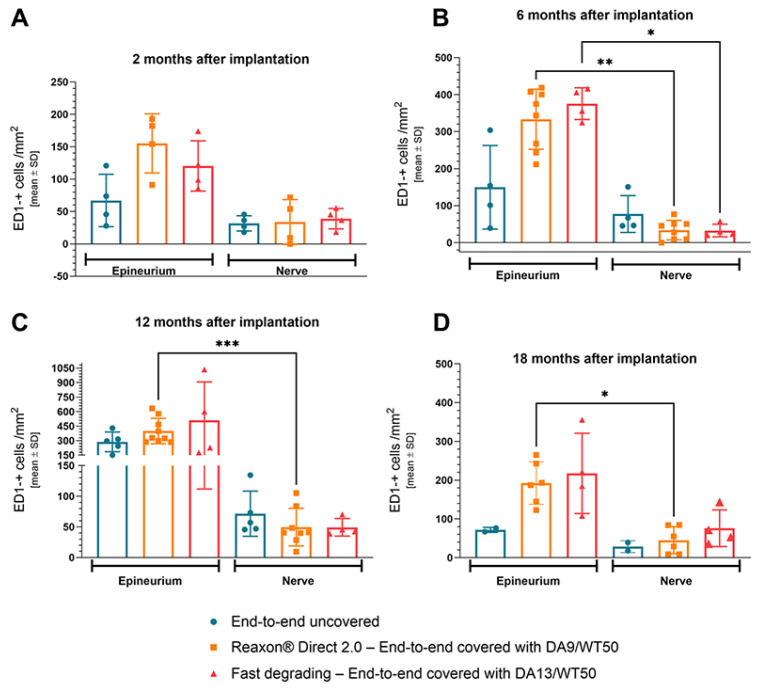
Quantitative assessment of ED1-immunopositve cells was performed in four regions of interest (20× magnification) in two cross sections per sample. Left bars depict results from analyzing the epineurium, right bars depict results from analyzing the nerve tissue area. (**A**) 2 months, (**B**) 6 months, (**C**) 12 months, and (**D**) 18 months after implantation, the results revealed a more pronounced presence of ED1-immunopositive macrophages in the epineurium compared to the nerve tissue. Each symbol represents the mean result derived from one sample. It is important to consider the different scaling of the y-axis. Data are expressed as a mean ± SD. Statistical analysis: Kruskal–Wallis-Test. * *p* ≤ 0.05; ** *p* ≤ 0.01, *** *p* ≤ 0.005.

Cross-sections through the nerve and its epineurium were incubated for the immunohistological detection of ED1-immunopositive cells, e.g., activated macrophages. Figure 4 depicts the results of the quantification over time. Representative photomicrographs analyzed at the respective time points are provided in the Supplementary Material (Figures S5–S8).

Two months after implantation, no statistically significant differences were detectable for the quantification of activated immune cells (ED1-immunopositive macrophages). However, it is obvious from Figure 4A, that activation is higher in the outer area (epineurium) than in the proper nerve tissue. ED1-immunopositive cells were detectable in both the uncovered as well as the CNC-covered samples (Figure S6). Therefore, it can be assumed that this finding indicates the normal immune reaction on foreign bodies, e.g., suture material or CNC. The higher amount of ED1-immunopositive cells in the epineurium may be attributed to the fact that the immune cells arrive from the outer tissue layers, or invade them more, and are therefore more attracted to the epineurium and less present in the nerve tissue.

Six months after implantation, it is noteworthy that the number of ED1-immunopositive cells detectable in the epineurium had almost doubled in comparison to values obtained at 2 months after implantation, whereas the changes in the proper nerve tissue were neglectable (Figure 4B, consider the scaling of the *Y*-axis). The increasing number of ED1-immunopositive cells between 2 and 6 months after implantation is most likely due to the degradation of the implanted CNCs, thus reflecting the typical immune response to the degrading foreign bodies. Interestingly, in comparison to the uncovered control end-to-end repaired samples, samples from CNC-covered repaired nerves showed an increase in immune reaction at the epineurium level, but a decrease at the nerve level from 2 to 6 months after implantation (Figure S7). The difference was also significant for both CNC-covered groups (DA9/WT50: *p* = 0.0023; DA13/WT50: *p* = 0.038). However, no significant differences were detected between the different groups at both investigated tissue areas.

Twelve months after implantation, no significant differences were detected between the groups in either the epineurium or the nerve tissue. However, it is obvious from Figure 4C, that activation in the outer area (epineurium) was again higher than in the nerve itself. This difference was also significant for the group with DA9/WT50-CNC covered end-to-end repair (*p* = 0.0006). As already detected in the previous time points, ED1 immunopositive cells were detectable in all groups (Figure S7). For the CNC-covered samples, the number of ED1-immunopositive cells detectable in the epineurium slightly increased further in comparison to the 6-month values, when it had almost doubled in comparison to the 2-month study. However, the numbers detectable in the epineurium of the uncovered samples increased from 6 to 12 months after implantation. Again, as after 6 months from surgery, there was no change detectable for the number of ED1-immunopositive cells in the proper nerve tissue.

Eighteen months after implantation, no statistically significant differences were detectable between the groups for the quantification of activated immune cells. Again, activation of macrophages was more pronounced in the epineurium than in the proper nerve tissue (Figure 4D). This difference was significant, however, only for the DA9/WT50 group (*p* = 0.0287), although ED1 immunopositive cells were detectable in all groups (Figure S8).

For the period from 6 to 12 months after surgery, there was a substantial increase detectable for the number of ED1 immunopositive cells in the epineurium. After 18 months, we detected a reduction to values rather similar to the 2-month data.

## 3. Discussion

One main goal of this study was to evaluate the degradation behavior of Reaxon^®^ Direct 2.0 (DA9/WT50) and a variant of it (DA13/WT50) in a preclinical setting.

For the Reaxon^®^ Direct 2.0 (DA9/WT50) our data confirmed stability and flexibility over 2 months. The variant DA13/WT50 demonstrated a considerable fragmentation already at 2 months after implantation with samples being almost completely fragmented into soft CNC pieces of variable size after 6 months.

With the extended observation time of 12 months post implantation, the DA13/WT50 material was much more deconstructed and occasionally found to be encapsulated ectopically to the original surgery site, while no CNC was observed anymore along the nerve. DA9/WT50 material demonstrated a certain degree of fragility, but a more preserved structural integrity as long as kept in situ at the site of surgery. However, with dissection, the material likewise fell apart into a variable number of CNC pieces of variable size.

Finally, a further progress in degradation of DA9/WT50 devices was observed after 18 months from surgery with, again, a preserved shape but a flattened structure in situ, indicating that the CNCs had broken into two longitudinal larger pieces, or even to be more fragile and broken up into much smaller pieces. Degradation of the DA13/WT50 devices was recorded to be further progressed at this later time point: the material was largely broken up into a high number of small pieces, which were mainly encapsulated in connective tissue around the original surgery site.

We demonstrated that degradation of both CNC variants resulted in the formation of the above mentioned CNC pieces, but at considerably different time points. These pieces were prone to be encapsulated at the outside of the nerve. As long as the structural integrity of the CNCs was mainly preserved in situ, encapsulated CNC material could be detected within the epineurium underneath the degrading CNC. When the structural integrity of the CNC was already dissolved, as predominantly seen in DA13/WT50 variants in our study, residual material from the CNCs was detected in soft ectopic connective tissue “pockets” with no direct contact to the nerve.

The second goal of our study was to evaluate immunological tolerance against Reaxon^®^ Direct 2.0 variants upon implantation and degradation. Our data confirm that, at 2 months and 6 months from implantation, the different Reaxon^®^ Direct 2.0 variants did not induce immunological tissue reactions, and that nerve regeneration was not negatively affected by any of the CNCs. An increase in epineurium thickness was detected also for the control condition, uncovered end-to-end-suture, and no differences between the groups was detectable. A slight increase in the number of ED1-immunopositive cells within the proper nerve tissue was detected between 6 and 12 months. We speculate that this was related to the progressive degradation of the implanted CNCs and represents the normal response of the immune system to the degrading foreign bodies. Until 18 months after implantation, the number of activated macrophages (ED1-immunopositive, [3,4]) decreased again. We assume that this was related to the further progress in CNC degradation and represents the normal reduction in immune activation with increasing efficiency in removing foreign material. Furthermore, we did not detect any multinucleated giant cells within the analyzed sections at 6 months, 12 months, or 18 months from surgery, indicating that degradation progresses without further challenge for the local immune response.

We consider that a higher amount of ED1-immunopositive cells within the epineurium indicated an increased activation of an immune response in comparison to the proper nerve tissue at all time points. The epineurium has further been found to enclose advanced CNC degradation products within pocket-like structures near the nerve. From a histological point of view, however, the nerve tissue itself remained unaffected by CNC degradation, and we also found no evidence of significant (critical) fibrosis at the CNC implantation sites. Therefore, from our pre-clinical study, it re-emerges that the different Reaxon^®^ Direct variants are basically well tolerated by the tissue and do not interfere with nerve regeneration in terms of the quantity of regenerated myelinated nerve fibers and the quality of their myelination.

The fact, however, that CNC pieces formed during the degradation process and appeared to be prone to be encapsulated in some vicinity to the original implantation side implies that these encapsulation events, depending on the implantation side, may become irritating to neighboring tissue. Indeed, it had already been reported from other biomaterials that degradation upon in-human use was not as expected from pre-clinical research and resulted in the need for removing surgeries [9,10,11]. While anticipation was rather high with regard to the performance of Reaxon^®^ products [2,5,6], it has to be noted that recent clinical reports indicate limitations for their in-human use as well [8,12].

In view of the preclinical findings that have been gathered in parallel to the clinical use of Reaxon^®^ Direct variants 1.0 and 2.0 [5,6], we have to consider that the recent reports of foreign body sensation by the patients and the necessity for removing surgeries from digital nerve repair sites, especially of Reaxon^®^ Direct 1.0, ref. [8] is maybe not that surprising. It further strengthens the importance and value of comprehensive pre-clinical evaluation of medicinal products, since pre-clinical studies prove to still be a valuable tool in translational research and are indeed quite predictive. Aman et al. link their clearly disappointing clinical experience to the slow degradability of the CNC material [8]. In view of our own experiences from the current study, rather we consider not the timing of degradation but the fate of the degrading material a serious concern, when it comes to the question of where to implant CNCs. The specific application on human digital nerves has to be considered less appropriate from the reports [8].

Our pre-clinical experiences, however, do not indicate that the chitosan-based CNC per se is not a valuable tool. Furthermore, at the same time, chitosan is still favored as a component in medicinal products for diverse treatments, including neuronal repair [1,13,14,15]. The most valuable combination with other biomaterials and also the most valuable specific design of the respective nerve conduits, however, is still under debate [8,16,17,18,19,20].

## 4. Materials and Methods

### 4.1. Animals and Surgery

In vivo experiments were performed under general anesthesia, Zolazepam (Zoletil, Virban) + Xilazine (Rompun, Bayer, Leverkusen, Germany) by intraperitoneal injection (40 mg/kg body weight + 5 mg/kg body weight), on a total of 38 adult female Wistar rats (Envigo, Udine, Italy), weighing approximately 200 g.

Transected median nerves were repaired with an end-to-end repair and wrapped with Reaxon^®^ Direct or one of its variants and harvested after 2 months, 6 months, 12 months, and 18 months, respectively. The treatment groups are reported in Table 1 above.

Once under proper general anesthesia, animals were shaved at the axillary region and the medial aspect of the upper forelimb and the region disinfected with Betadine (Meda Pharma S.p.A., Mailand, Italy). All surgical procedures were performed under a sufficiently high magnification by a surgical microscope (TAKAGIOM-08 (Takagi, Nagano-ken, Japan)), ensuring sterile procedures in a clean room.

All CNCs were immersed in sterile saline for at least 10 min before implantation. In all animals, both median nerves were approached from the axillary region to the elbow; the nerves were transected and immediately repaired with an end-to-end repair by a 9/0 non-absorbable suture (PVDF, Peters Surgical Premio #26S03B (Boulogne-Billancourt, France)). A 10 mm long chitosan nerve conduit (CNC) was pulled over the point of end-to-end suture and anchored to both nerve stumps with one additional suture to the epineurium. For enabling this procedure, one transected nerve end was inserted into the CNC before nerve repair. At the end of the surgical procedure the skin was sutured by a 3/0 single non-absorbable suture (PVDF, Peters Surgical Premio # 26S20C).

At day 0, 1, 2, and 3 post-surgery, an analgesic therapy (Rimadyl^®^, 4 mg/kg body weight, Zoetis Italia) was administered by subcutaneous injection, while at day 2 and 5 post-surgery, an antibiotic treatment (Rubrocillina 0.05 mL/500 g body weight, MSD animal health) was administered by intramuscular injection. During the post-implantation period, the general health status of the rats was evaluated considering the following parameters: shine of the rat fur, reactivity, physiological habitus, and aspect of the surgical wound (normal healing/slight inflammation/inflammation). Animals were observed at day 0 before surgery, at day 1, 2, and 3 post-surgery, and once per month thereafter. Weight evolution of the animals was also recorded at day 0 and at each day of final sample harvest at month 2, at month 6, at month 12, or at month 18, respectively (last day of procedure). No sudden decreases in weight were observed and, throughout the experimentation period, animals displayed a physiological increase in body mass (except for between 12 and 18 months where animals’ weight remained approximately stable).

After 2 months, 6 months, 12 months, or 18 months, rats were put under general anesthesia as above for sample harvest and immediately thereafter killed through an anaesthetic overdose of Zoletil + Xilazine (>60 mg/kg bodyweight and >10 mg/kg bodyweight, respectively) administered likewise by intraperitoneal injection.

### 4.2. Fabrication of Reaxon^®^ Direct Variants

The chitosan material was derived from Pandalus borealis shrimp shells and processed to medical grade by Chitinor AS (Tromsø, Norway). Chitosan tubes were manufactured by Medovent GmbH (Mainz, Germany) under ISO 13485 conditions from chitin tubes of different wall thicknesses (WT) made by a proprietary extrusion process, followed by distinctive washing and hydrolysis steps to adjust the required degree of acetylation (DA). Chitosan tube variants of DA5/WT100, DA9/WT50 and DA13/WT50 have therefore been manufactured. Tubes were finally cut into the required lengths and sterilized by ethylene oxide.

### 4.3. Macroscopic Assessment of the Implantation Site upon Sample Harvest

Upon harvest, after 2 months, 6 months, 12 months, or 18 months, each specimen was photographed and then divided into CNCs (as far as biodegradation allowed for), proximal and distal segment of the nerve. The CNCs underwent specific visual control: transparency, signs of degradation, like cracks, brittleness, decomposition in pieces, and habit of the same.

### 4.4. Histological Analysis of the Nerve Tissue

#### 4.4.1. Resin Embedding and Stereomorphometrical Analysis

The stereology study of myelinating nerves to evaluate the nerve regeneration process is performed, as extensively described in [21], and here briefly reported. The distal part of the nerve samples was fixed by immediate immersion in 2.5% glutaraldehyde (SIC Società Italiana Chimici, Rome, Italy, #16210) in 0.1 M phosphate buffer (pH 7.4) for 5–6 h at 4 °C. Samples were then post-fixed in 2% osmium tetroxide (SIC Società Italiana Chimici, Rome, Itlay, #19170) for 2 h and dehydrated in passages in ethanol (Sigma Aldrich, Milan, Italy, #32221) from 30% to 100% (5 min each passage). After two passages of 7 min in propylene oxide and overnight in a 1:1 mixture of propylene oxide (Sigma Aldrich #110205) and Glauerts’ mixture of resins, samples were embedded in Glauerts’ mixture of resins made of equal parts of Araldite M (Sigma Aldrich, Milan, Italy, #10951) and the Araldite Harter, HY 964, (Sigma Aldrich, Milan, Italy, #45346). In the resin mixture, 0.5% of the plasticizer dibutylphthalate (Sigma Aldrich, Milan, Italy, #12487) was added. For the final step, 2% of accelerator 964 DMP-30 (Electron Microscopy Sciences, Rome, Italy, #13600) was added to the resin in order to promote the polymerization of the embedding mixture, at 60 °C.

Semi-thin transversal sections (2.5 µm) were cut using an Ultracut UCT ultramicrotome (Leica Microsystems, Wetzlar, Germany) and stained with 1% toluidine blue for high resolution light microscopy examination and design-based stereology. A DM4000B microscope equipped with a DFC320 digital camera, and an IM50 image manager system (Leica Microsystems, Wetzlar, Germany) was used for section analysis.

Stereological analysis was performed on 2.5 µm toluidine blue stained sections with light microscopy and the following parameters were estimated: cross sectional area [mm^2^], density of myelinated nerve fibers/mm^2^, number of myelinated fibers, axon diameter of myelinated fibers [µm], fiber diameter [µm], myelin thickness [µm], axon diameter/fiber diameter (*g*-ratio). The previously mentioned parameters are indicators for the success of axonal regeneration after nerve transection and the respective end-to-end repair. Briefly, the nerve cross-sectional area provides an overall view of nerve growth and regeneration, indicating whether the nerve has regained its original size and structure after injury. Stereological parameters, such as the number and density of myelinated fibers, are key indicators of the quality and extent of nerve regeneration, but these data are typically considered alongside size parameters for a more complete assessment. Axon diameter, fiber diameter, and myelin thickness are critical for evaluating the functionality of nerve fibers and the degree of myelination, which is necessary for restoring proper nerve function after injury. Similarly, the g-ratio, which compares axon diameter to fiber diameter, is an important measure for assessing the efficiency of nerve signal conduction. It helps determine whether the degree of myelination is appropriate for the axon size, as it correlates with fiber maturation and conduction speed. Together, these parameters provide a comprehensive evaluation of nerve regeneration, reflecting both structural and functional recovery. They allow for an assessment of how well the nerve has healed, how effectively it can conduct signals, and whether it has regained its ability to support normal function after injury [22].

#### 4.4.2. Paraffin Embedding

The nerve segments comprising the end-to-end suture (either uncovered or previously covered by CNCs) plus 3–4 mm in proximal and distal direction, were harvested and fixed overnight at 4 °C in 4% paraformaldehyde (PFA; Sigma-Aldrich Chemie GmbH, Schnelldorf, Germany) diluted in phosphate buffered saline (PBS; Dulbecco, Biochrom GmbH, Berlin, Germany). Afterwards, the samples were stored in 70% ethanol prior to paraffin-embedding in a Citadel tissue embedding-automatic unit (Shandon Citadel 2000, Fisher Scientific, Pittsburgh, PA, USA). For histological analysis of the nerve and its epineurium, blind-coded 7 µm paraffin sections were cut from the distal end of the nerve samples.

#### 4.4.3. Giemsa Staining

Immunocytochemistry was performed on two deparaffinized cross sections (approx. 70 µm apart from each other in distal-proximal direction). For this, sections were incubated for 2 h in 2% Azure eosin methylene blue, Giemsa stain modified solution (#32284, Sigma Aldrich, Darmstadt, Germany) diluted in distilled water (dH_2_O). This was followed by a 2 sec-incubation with dH_2_O added with 2 drops of acetic acid. The sides with the sections were then passed into 100% isopropyl alcohol (J.T.Baker^®^, Fisher Scientific, Darmstadt, Germany) and xylene (J.T.Baker^®^, Fisher Scientific, Germany) for 30 sec each. Finally, the sections were covered with a Eukitt^®^ mounting medium (Merck, Darmstadt, Germany) and covered with a glass slip. Light microscopic (Olympus BX53 and Olympus BX51, Soborg, Denmark) evaluation of Giemsa stained sections was aided by the programs cellSens Dimensions and cellSens Entry (both Olympus; Soborg, Denmark). Furthermore, the number of multinucleated giant cells (MGC) per mm^2^, as well as the number of chitosan inclusions inside the connective tissue sections, was manually quantified by the examiner (blinded to the experimental condition).

In two Giemsa stained sections per sample, the epineurial thickness was determined at 4 randomly chosen points and from this a mean was calculated for each sample. Single sample means were added on for calculating the mean ± standard deviation for each group.

#### 4.4.4. Immunofluorescence Against ED1

Immunohistology was performed to estimate the number of ED1-stained cells, i.e., activated macrophages. For this, two blind-coded 7 mm paraffin cross sections (approx. 70 µm apart from each other in distal-proximal direction), consecutive to the Giemsa stained sections were de-paraffinized. Then the sections were incubated in PBS containing 5% rabbit serum as a blocking step prior to incubation with primary mouse anti-ED-1 antibody (1:1000; MCA 275R Serotec, Oxford, UK) at 4 °C overnight. The next day sections were washed with PBS before being incubated with Alexa 555-conjugated secondary goat-anti-mouse antibody (1:1000; A21422, Invitrogen, Darmstadt, Germany) for 1 h at room temperature. Sections were finally counterstained with the nuclear dye 40,60-diamino-2-phenylindole (DAPI, 1:2000, Sigma-Aldrich, Schnelldorf, Germany) in PBS for 2 min at room temperature and then mounted with Moviol (Calbiochem, Darmstadt, Germany, N 475904). Six photomicrographs were randomly taken from each section (20× magnification), each for the epineurial area and for the nerve area, using an IX70 microscope (Olympus) and the cellP software 5.0 (Olympus, Soborg, Denmark) (Olympus). The number of ED1-positive cells was calculated as number/mm^2^ with the help of the ImageJ software 1.53 (NIH, Bethesda, MD, USA). The nuclear DAPI counterstaining was used to clearly identify ED1-immunopositive cells [4].

### 4.5. Statistical Analysis

Statistical analysis was performed using Graph Pad Prism 8.0 Software for windows (Graphpad Software, Boston, MA, USA). Statistical analysis was performed using one-way ANOVA followed by Bonferroni’s post-hoc or *t*-test, Kruskal–Wallis test for multiple comparison of histological data. The level of significance was set at *p* ≤ 0.05 (*), *p* ≤ 0.01 (**), and *p* ≤ 0.001 (***). Values are expressed as a mean ± standard deviation (SD) throughout the manuscript.

## 5. Conclusions

In conclusion, the recent literature available for the clinical use of Reaxon^®^ Direct and the novel pre-clinical data reported here, should increase the critical appreciation of careful preclinical evaluation and their predictive value for a specific clinical scenario. Furthermore, we demonstrate that the pre-clinical use of chitosan-based nerve conduits does not negatively interfere with the nerve regeneration and reports exist on positive effects, also in human applications [5]. Therefore, we still consider that existing CNCs could be a valuable tool for supporting regeneration after end-to-end repair, but should be applied only in locations where encapsulation of the degrading material would have more space and be much less likely to evoke unpleasant and disturbing foreign-body-sensations.

## Figures and Tables

**Table 1:** Experimental groups and animal numbers originally included in this study. N—number of animals introduced per group.

Group 1	2 Months	6 Months	12 Months	18 Months
Right median nerve: End-to-end uncovered	N = 4	N = 4	N = 4	N = 6
Left median nerve: Reaxon^®^ Direct 2.0—End-to-end covered with DA9/WT50				
**Group 2**				
Right median nerve: Fast degrading—End-to-end covered with DA13/WT50	N = 4			
**extended Group 2**				
Right median nerve: Fast degrading—End-to-end covered with DA13/WT50		N = 4	N = 5	N = 6
Left median nerve: Reaxon^®^ Direct 2.0—End-to-end covered with DA9/WT50				

**Table 2:** Number of specimens analyzed per condition.

	2 Months	6 Months	12 Months	18-Month
End-to-end uncovered (control)	N = 3	N = 4	N = 5	N = 2
Reaxon^®^ Direct 2.0—End-to-end covered DA9/WT50	N = 4	N = 8	N = 9	N = 5
Fast degrading— End-to-end covered DA13/WT50	N = 4	N = 4	N = 4	N = 4

## Data Availability

The raw data supporting the conclusions of this article will be made available by the study PI, G.R.—corresponding author, on request.

## Supplementary Materials

The following supporting information can be downloaded at: https://www.mdpi.com/article/10.3390/ijms26031214/s1.
