# Supplementary material for: Chitosan-Based Materials for Peripheral Nerve Repair—New Pre-Clinical Data on Degradation Behavior at the Nerve Repair Site and Critical Opinion on Their Translational Impact
Source: Int J Mol Sci. 2025 Jan 30;26(3):1214. doi: 10.3390/ijms26031214 (PMC11818691; doi:10.3390/ijms26031214)
Supplement: Supplementary file 1 [file ijms-26-01214-s001.zip › ijms-3410238-supplementary.pdf]

Chitosan-based materials for peripheral nerve repair - new pre-clinical data on degradation behavior at the nerve repair site and critical opinion on their translational impact

Giulia Ronchi [1,] *, Christina Ackva [2,3] , Federica Fregnan [1], Federica Zen [1], Luisa Muratori [1], Alessandro Crosio [4], Jennifer Metzen [5] and Kirsten Haastert-Talini [5,6]

1 Department of Clinical and Biological Sciences & Neuroscience Institute Cavalieri Ottolenghi (NICO), University of Torino, Orbassano, 10043, (Torino), Italy; giulia.ronchi@unito.it; federica.fregnan@unito.it; luisa.muratori@unito.it; federica.zen@unito.it;
2 KeriMedical, Route des Acacias, 45a, 1227 Genf, Switzerland
3 Medovent GmbH, Friedrich-Koenig-Str. 3, 55129 Mainz, Germany; mail@medovent.com
4 UOC Traumatology-Reconstructive Microsurgery, Department of Orthopedics and Traumatology, CTO Hospital, Turin, Italy; alessandro.crosio@unito.it
5 Institute of Neuroanatomy and Cell Biology, Hannover Medical School, Carl-Neuberg-Str.1, 30625 Hannover, Lower-Saxony, Germany
6 Centre for Systems Neuroscience (ZSN), Hannover, 30559 Hannover, Lower-Saxony, Germany
* Correspondence: giulia.ronchi@unito.it

Supplementary Materials:

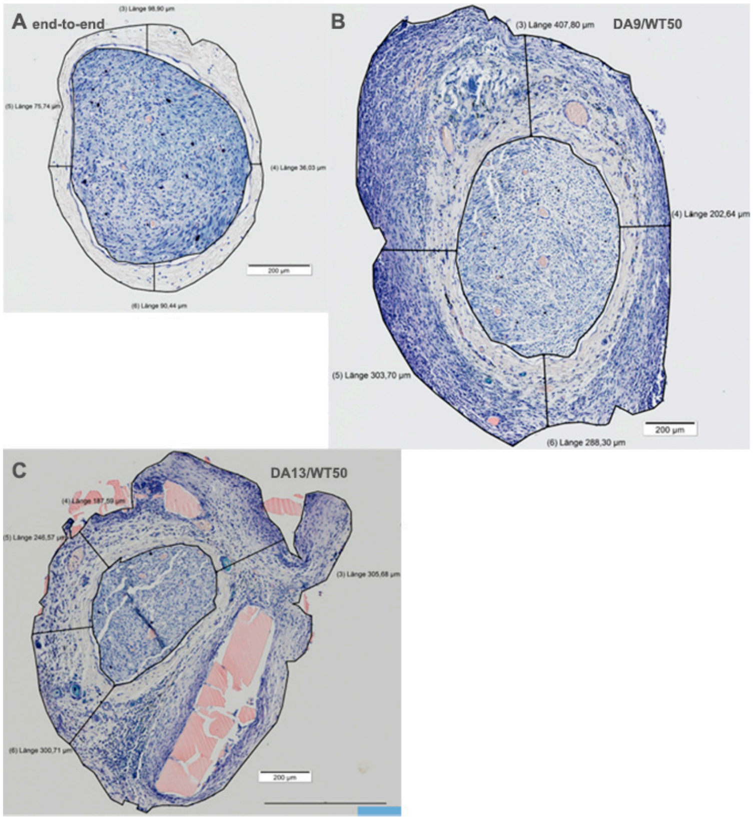

Figure S1: Van Giemsa-stained cross sections through the nerve and its epineurium at 2-months after implantation. (A) End-to-end repair uncovered; (B) End-to-end repair covered with chitosan nerve conduit (CNC) variant DA9/WT50; (C) End-to-end repair covered with CNC variant DA13/WT50. Degrading chitosan material is detectable as pinkish amorphous material within the epineurium. Numbered lines represent the location of measured epineurial thickness (Länge = length in µm). The

inner encircled area represents the nerve, the outer encircled area represents the outer border of the epineurium as it was dissected from the implantation side (uncovered nerve) or from within the CNC, respectively. Scale bars: 200 µm.

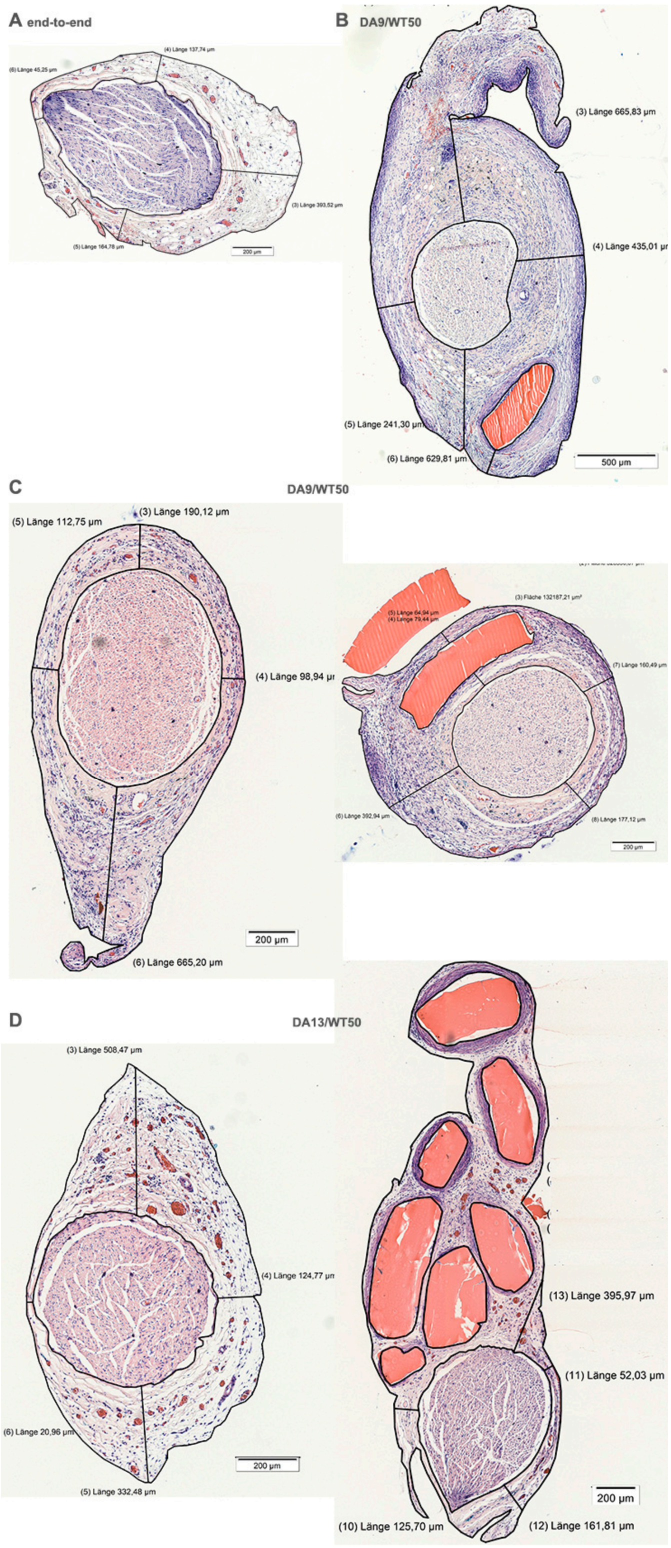

Figure S2: Van Giemsa-stained cross sections through the nerve and its epineurium at 6-months after implantation. (A) End-to-end repair uncovered; (B, C) End-to-end repair covered with chitosan nerve conduit (CNC) variant DA9/WT50, three different speciment; (D) End-to-end repair covered with CNC variant DA13/WT50, two different specimen. Degrading chitosan material is detectable as pinkish amorphous material within the epineurium. Numbered lines represent the location of measured

epineurial thickness (Länge = length in µm). The inner encircled area represents the nerve, the outer encircled area represents the outer border of the epineurium as it was dissected from the implantation side (uncovered nerve) or from within the CNC, respectively. Scale bars: 200 µm.

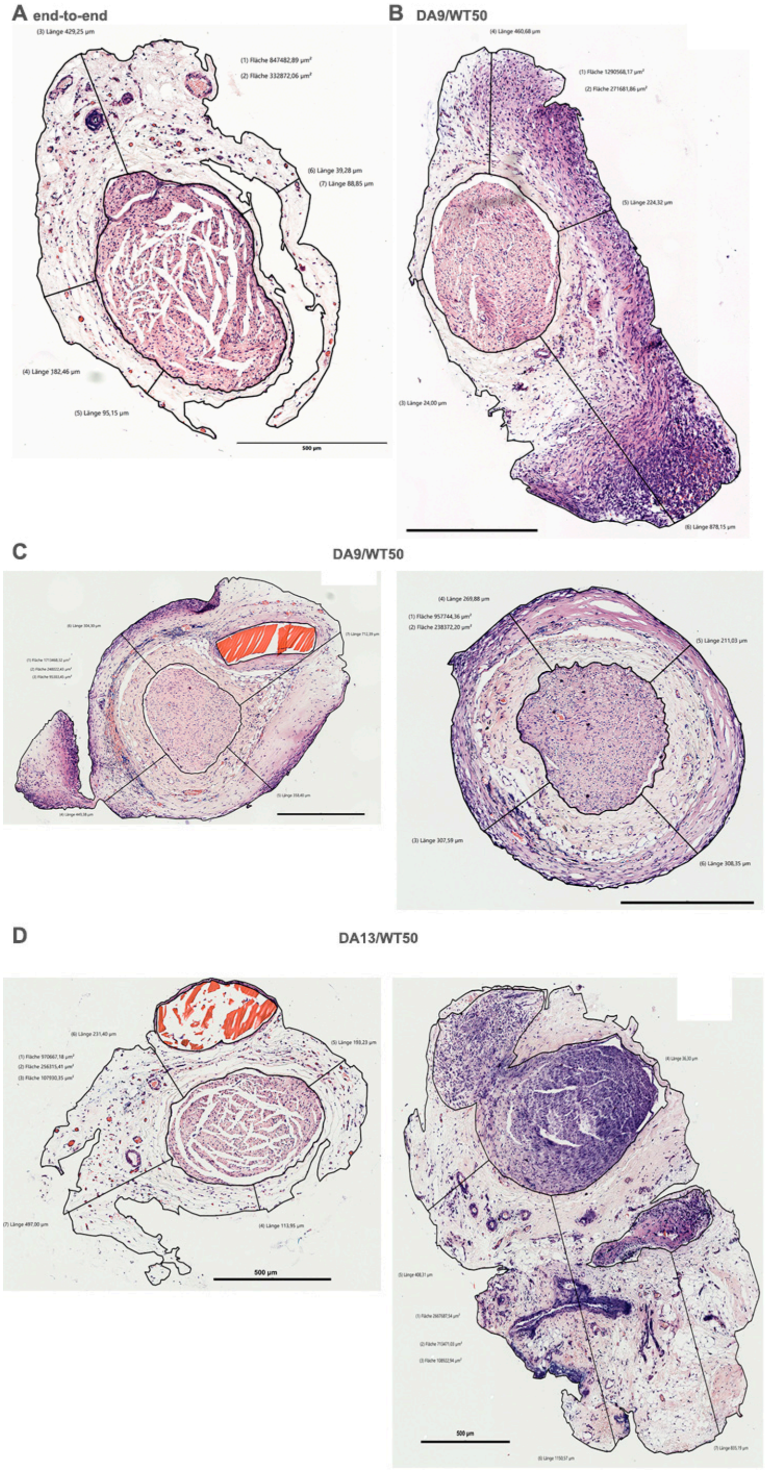

Figure S3: Van Giemsa-stained cross sections through the nerve and its epineurium at 12-months after implantation. (A) End-to-end repair uncovered; (B, C) End-to-end repair covered with chitosan nerve conduit (CNC) variant DA9/WT50, three different speciment; (D) End-to-end repair covered with CNC variant DA13/WT50, two different specimen. Degrading chitosan material is detectable as pinkish amorphous material within the enpineurium. Numbered lines represent the location of measured epineurial thickness (Länge = length in µm). The inner encircled area represents the nerve, the outer

encircled area represents the outer border of the epineurium as it was disected from the implantation side (uncovered nerve) or from within the CNC, respectively. Scale bars: 500 µm.

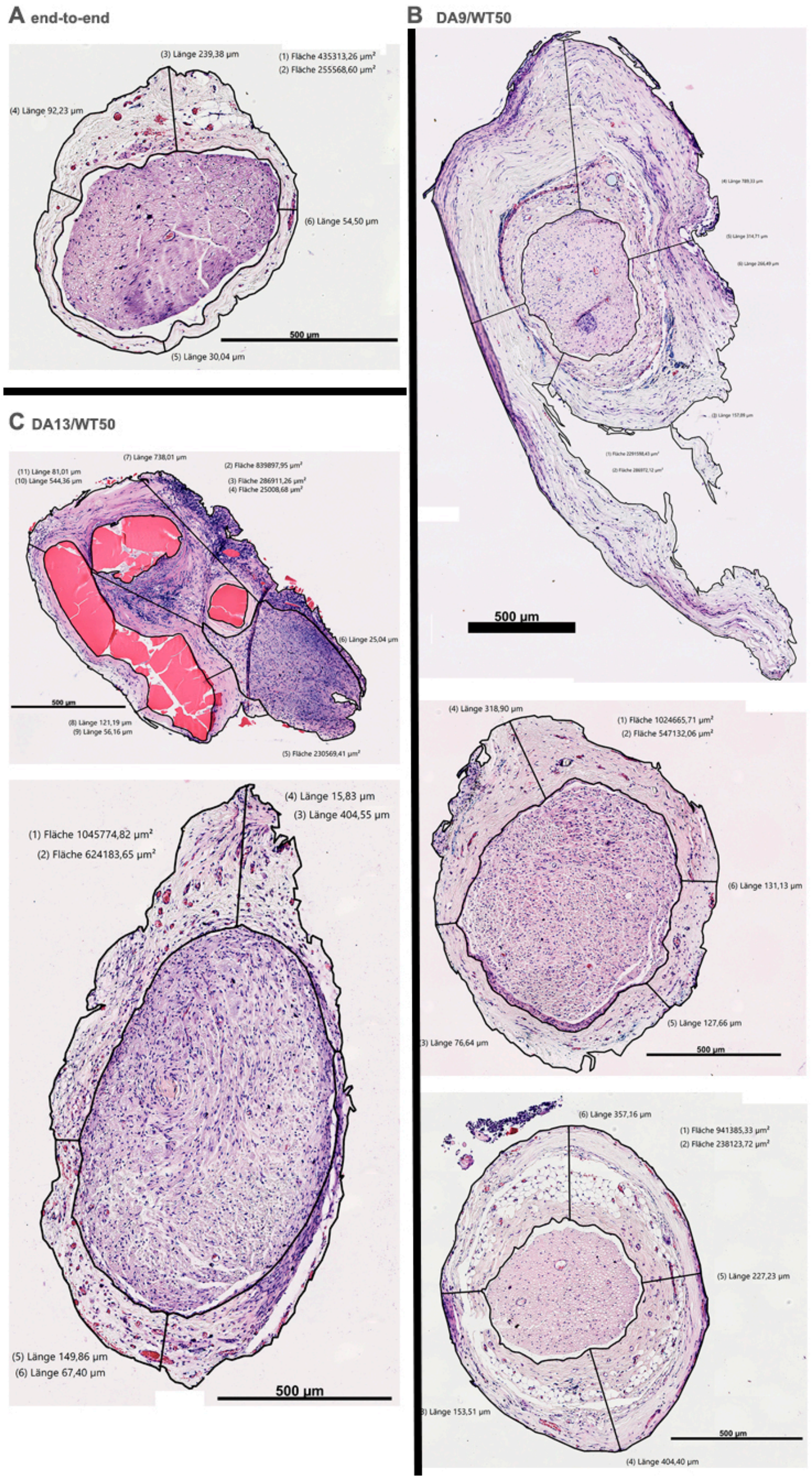

Figure S4: Van Giemsa-stained cross sections through the nerve and its epineurium at 18-months after implantation. (A) End-to-end repair uncovered; (B) End-to-end repair covered with chitosan nerve conduit (CNC) variant DA9/WT50, three different speciment; (C) End-to-end repair covered with CNC variant DA13/WT50, two different specimen. Degrading chitosan material is detectable as pinkish amorphous material within the epineurium. Numbered lines represent the location of measured epineurial thickness (Länge = length in µm). The inner encircled area repesents the nerve, the outer encircled area represents the outer border of the epineurium as it was disected from the implantation side (uncovered nerve) or from within the CNC, respectively. Scale bars: 500 µm.

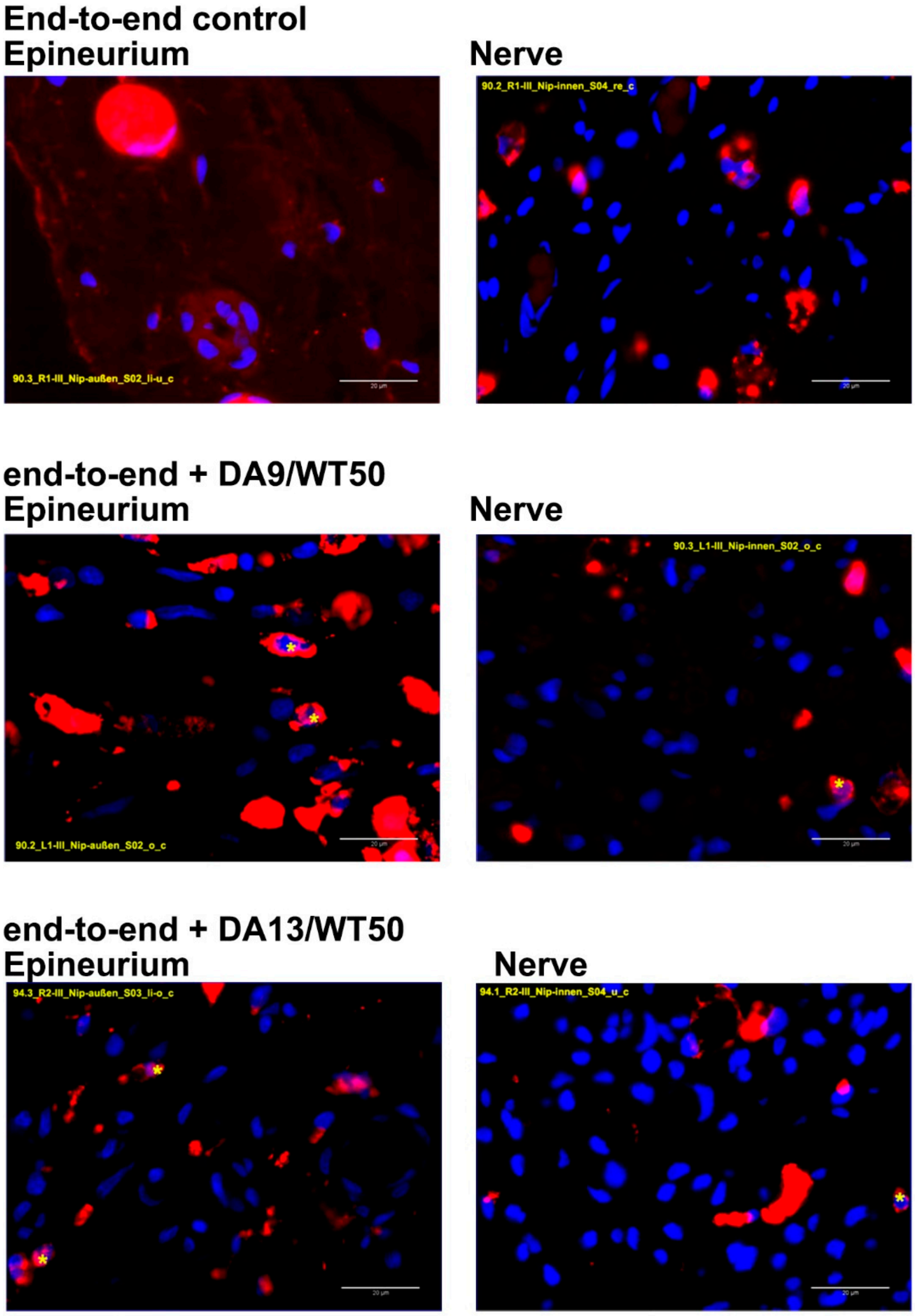

Figure S5: Representative details of ED1-immunopositive cells at 2-months after implantation. Qunatification was performed in randomly chosen regions of interest (20 x magnifications) in cross sections through the epineurium and the nerve itself. Left column shows photomicrographs from the epineurium. Right column shows photomicrographs from the proper nerve tissue. Upper row shows examples from End-to-end repair. Middel row shows examples from end-to-end repair covered by DA9/WT50-chitosan nerve conduit (CNC). Lower row shows examples from end-to-end repair covered by DA13/WT50-CNC. Yellow stars mark cells that were detected to be positivley stained for the ED1-antigen (red), counterstained nuclei are displayed in blue. Positive events were only counted, when the ED1-signal could undoubtely be correlated to a counterstained nucleus. Scale bars: 20 µm.

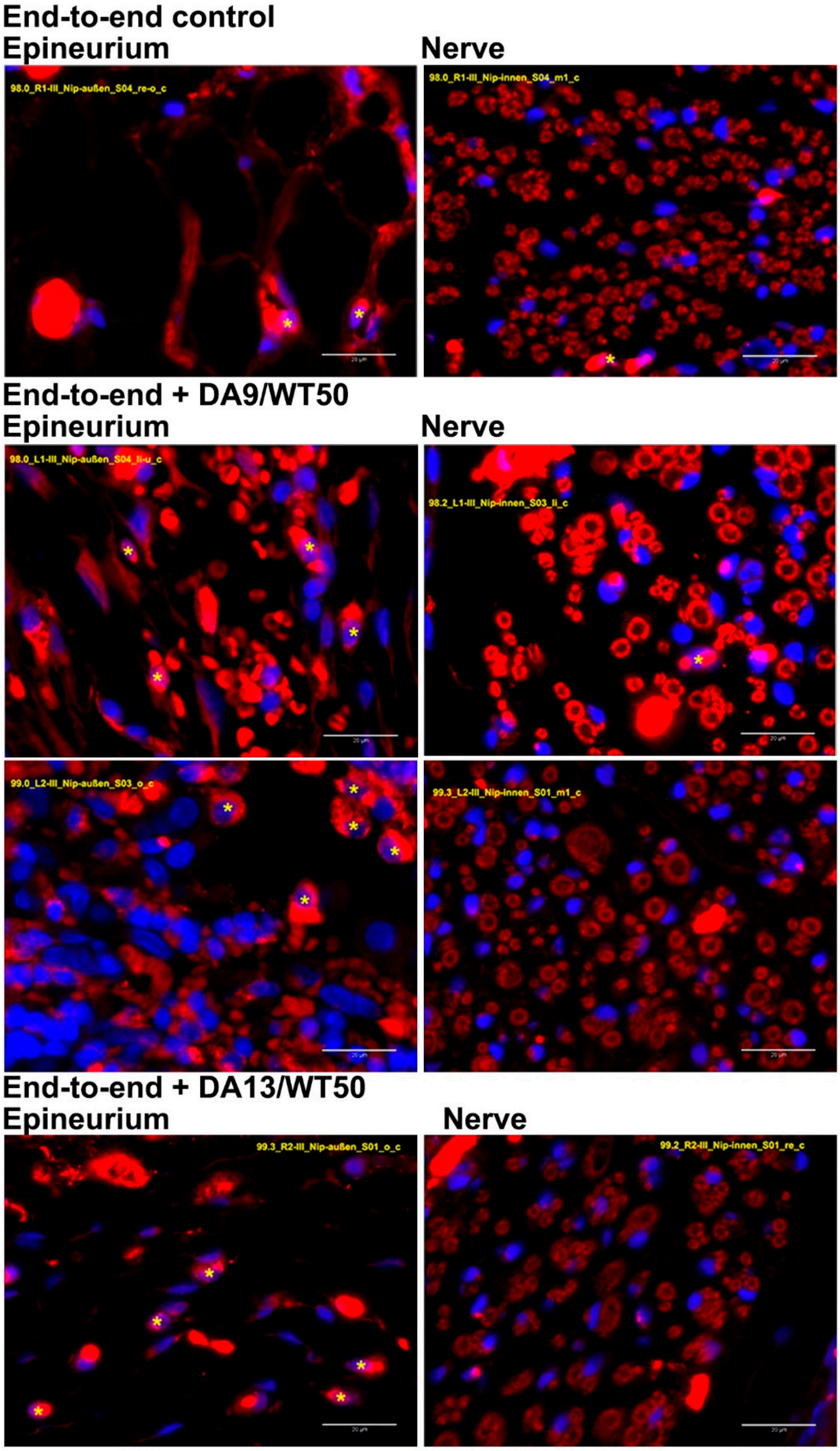

Figure S6: Representative details of ED1-immunopositive cells at 6-months after implantation. Qunatification was performed in randomly chosen regions of interest (20 x magnifications) in cross sections through the epineurium and the nerve itself. Left column shows photomicrographs from the epineurium. Right column shows photomicrographs from the proper nerve tissue. Upper row shows examples from End-to-end repair. Two middel rows show examples from end-to-end repair covered by DA9/WT50-chitosan nerve conduit (CNC). Lower row shows examples from end-to-end repair covered by DA13/WT50-CNC. Yellow stars mark cells that were detected to be positivley stained for the ED1-antigen (red), counterstained nuclei are displayed in blue. Positive events were only counted, when the ED1-signal could undoubtely be correlated to a counterstained nucleus. Scale bars: 20 µm.

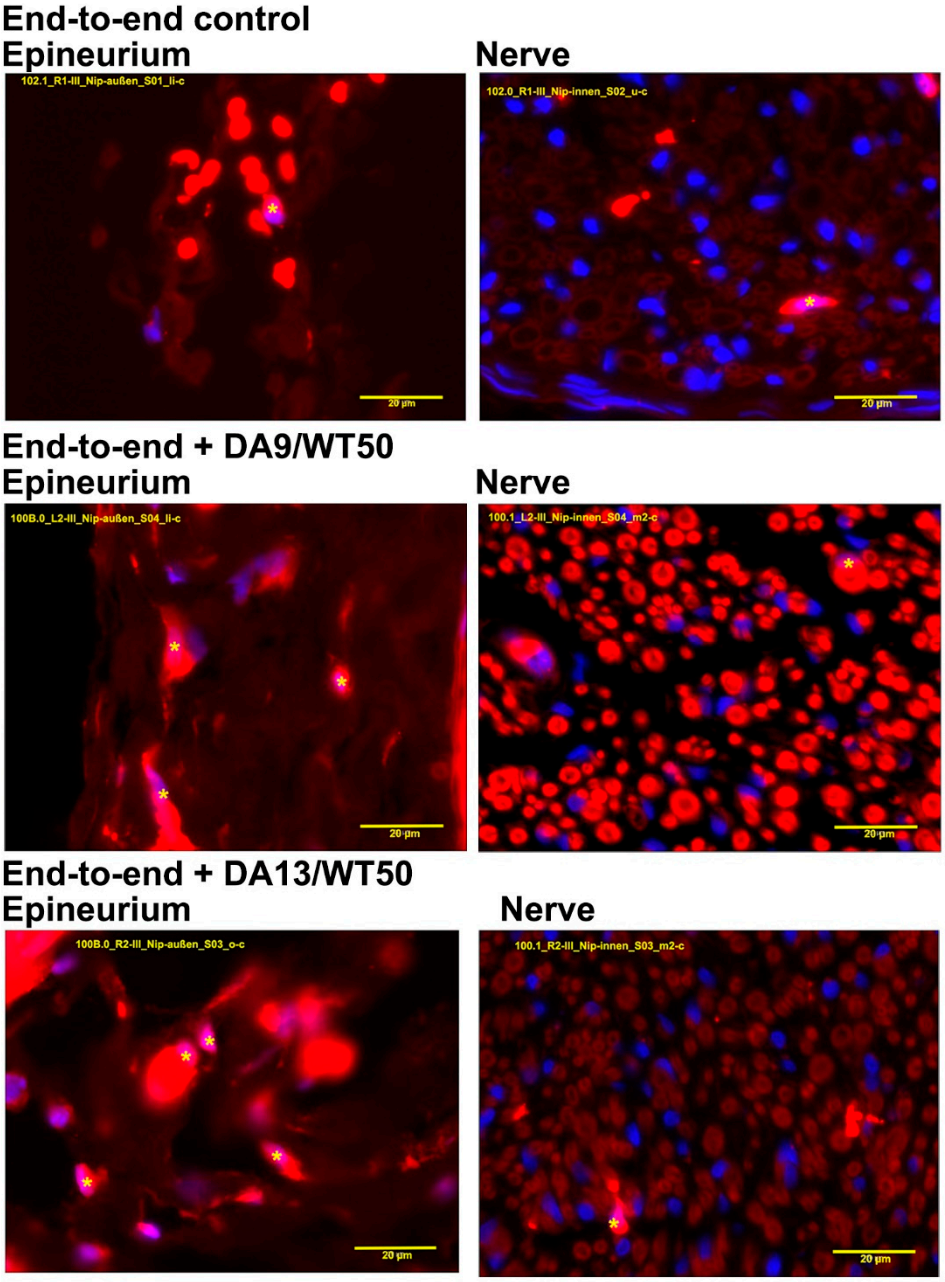

Figure S7: Representative details of ED1-immunopositive cells at 12-months after implantation. Qunatification was performed in randomly chosen regions of interest (20 x magnifications) in cross sections through the epineurium and the nerve itself. Left column shows photomicrographs from the epineurium. Right column shows photomicrographs from the proper nerve tissue. Upper row shows examples from End-to-end repair. Middel row shows examples from end-to-end repair covered by DA9/WT50-chitosan nerve conduit (CNC). Lower row shows examples from end-to-end repair covered by DA13/WT50-CNC. Yellow stars mark cells that were detected to be positivley stained for the ED1-antigen (red), counterstained nuclei are displayed in blue. Positive events were only counted, when the ED1-signal could undoubtely be correlated to a counterstained nucleus. Scale bars: 20 µm.

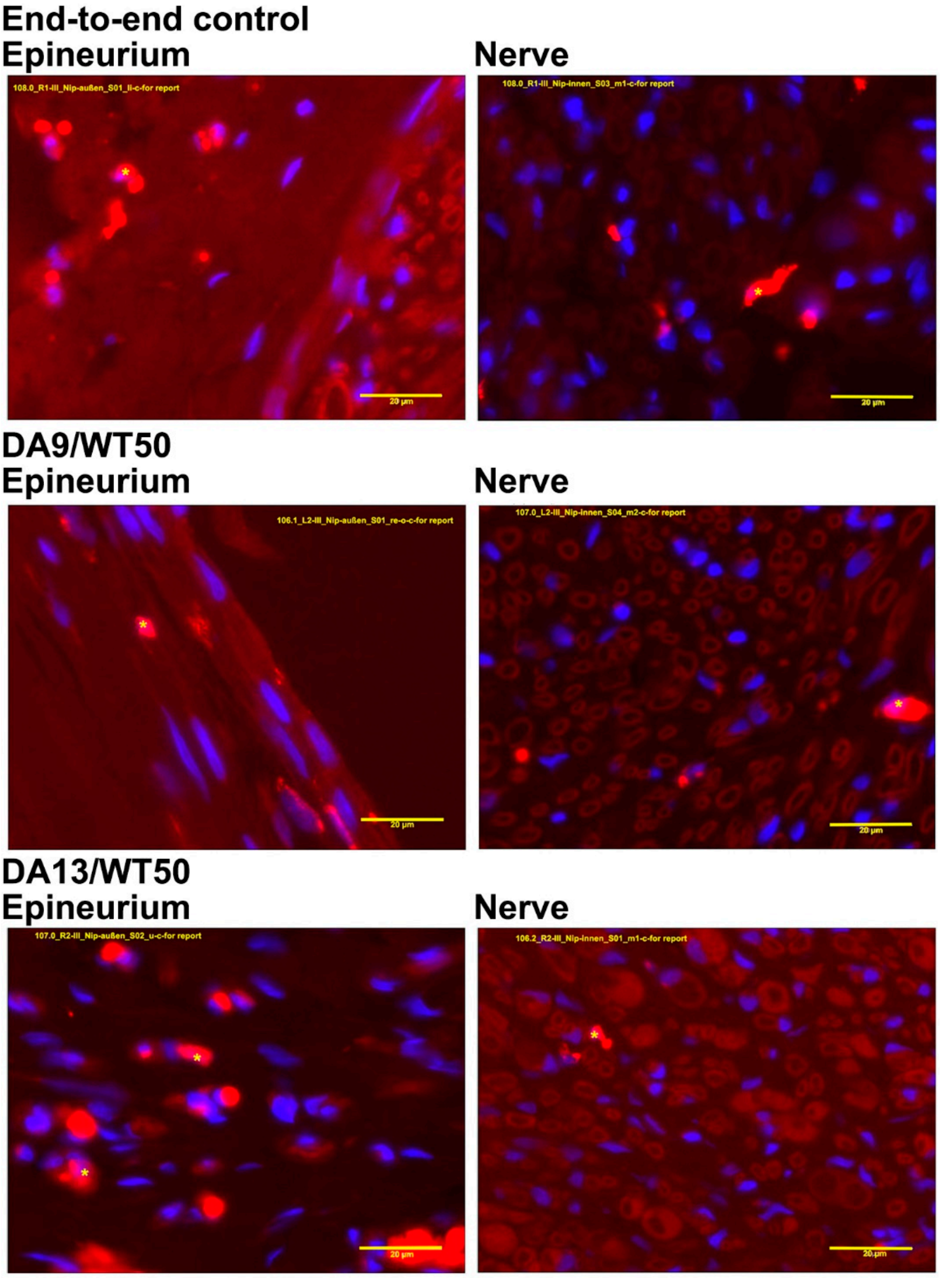

Figure S8: Representative details of ED1-immunopositive cells at 2-months after implantation. Qunatification was performed in randomly chosen regions of interest (20 x magnifications) in cross sections through the epineurium and the nerve itself. Left column shows photomicrographs from the epineurium. Right column shows photomicrographs from the proper nerve tissue. Upper row shows examples from End-to-end repair. Middel row shows examples from end-to-end repair covered by DA9/WT50-chitosan nerve conduit (CNC). Lower row shows examples from end-to-end repair covered by DA13/WT50-CNC. Yellow stars mark cells that were detected to be positivley stained for the ED1-antigen (red), counterstained nuclei are displayed in blue. Positive events were only counted, when the ED1-signal could undoubtely be correlated to a counterstained nucleus. Scale bars: 20 µm.
